# Identificación molecular de *Entamoeba histolytica, Entamoeba dispar* y *Entamoeba moshkovskii* en niños con diarrea en Maracaibo, Venezuela

**DOI:** 10.7705/biomedica.5584

**Published:** 2021-05-31

**Authors:** Zulbey Rivero, Lisbeth Villarreal, Ángela Bracho, Carem Prieto, Rafael Villalobos

**Affiliations:** 1 Laboratorio de Parasitología, Universidad del Zulia, Maracaibo, Venezuela Universidad del Zulia Laboratorio de Parasitología Universidad del Zulia Maracaibo Venezuela; 2 Laboratorio de Parasitología, Universidad Técnica de Manabí, Portoviejo, Ecuador Universidad Técnica de Manabí Laboratorio de Parasitología Universidad Técnica de Manabí Portoviejo Ecuador; 3 Laboratorio Clínico del Servicio Autónomo, Hospital Universitario de Maracaibo, Maracaibo, Venezuela Hospital Universitario de Maracaibo Laboratorio Clínico del Servicio Autónomo Hospital Universitario de Maracaibo Maracaibo Venezuela; 4 Departamento de Investigación, Universidad Católica de Cuenca, Cuenca, Ecuador Universidad Católica de Cuenca Departamento de Investigación Universidad Católica de Cuenca Cuenca Ecuador; 5 Centro de Investigaciones Endocrino-Metabólicas "Dr. Félix Gómez", Universidad del Zulia, Maracaibo, Venezuela Universidad del Zulia Centro de Investigaciones Endocrino-Metabólicas "Dr. Félix Gómez" Universidad del Zulia Maracaibo Venezuela

**Keywords:** Entamoeba, Entamoeba histolytica, reacción en cadena de la polimerasa multiplex, niño, diarrea, Venezuela, Entamoeba, Entamoeba histolytica, multiplex polymerase chain reaction, child, diarrea, Venezuela

## Abstract

**Introducción.:**

Las amebas no patógenas *Entamoeba dispar, Entamoeba moshkovskii* y *Entamoeba bangladeshi* son morfológicamente idénticas a *Entamoeba histolytica,* parásito responsable de la amebiasis, por lo cual se necesitan técnicas moleculares para diferenciarlas.

**Objetivo.:**

Determinar la frecuencia de las diferentes especies de *Entamoeba* mediante reacción en cadena de la polimerasa *(Polymerase Chain Reaction,* PCR) en muestras fecales de niños menores de cinco años con diarrea, provenientes de Maracaibo (Venezuela).

**Materiales y métodos.:**

Se recolectó una muestra fecal por individuo en 75 niños con diarrea (grupo de casos) y en 25 niños sin diarrea (grupo control). Las heces se evaluaron mediante examen microscópico, método de concentración de formól-éter y PCR múltiple anidada en una sola ronda para identificar *E. histolytica, E. dispar* y *E. moshkovskii.* Además, se hizo una encuesta en la que se recopilaron los datos demográficos, signos, manifestaciones clínicas y estrato socioeconómico de los niños.

**Resultados.:**

El 48 % de los participantes (38 del grupo de casos y 10 del grupo de control) tenían enteroparásitos. Solo en las muestras de cuatro de los niños, se encontraron quistes del complejo *Entamoeba* (tres en el grupo de casos y uno en el de control). Mediante PCR se amplificaron nueve muestras (9 %) para la detección de las amebas estudiadas. En el grupo de casos se registraron tres (28,13 %) de *E. histolytica,* cuatro (30,50 %) de *E. dispar* y una (9,37 %) de *E. moshkovskii,* en tanto que solo una (25 %) muestra amplificó para *E. dispar* en el grupo de control.

**Conclusión.:**

En general, predominó *E. dispar;* sin embargo, todos los infectados con *E. histolytica* se detectaron en el grupo de niños con diarrea y se detectó el primer caso de *E. moshkovskii* en la región.

## Abstract (en)

**Introduction:**

*Entamoeba histolytica* is an amebiasis-producing parasite. However, *Entamoeba dispar, Entamoeba moshkovskii,* and *Entamoeba bangladeshi* are non-pathogenic amoebae morphologically identical to it and, therefore, molecular techniques are required for their differentiation.

**Objective:**

To determine the frequency of *Entamoeba* species by polymerase chain reaction (PCR) in fecal samples from children under five years with diarrhea from Maracaibo (Venezuela).

**Materials and methods:**

A fecal sample per individual was collected from 75 children with diarrhea (case group) and 25 children without diarrhea (control group). Stools were evaluated by microscopic examination, formol-ether concentration method, and nested-multiplex PCR in a single round for the identification of *E. histolytica, E. dispar,* and *E. moshkovskii.* In addition, a survey was conducted in which demographic data, signs, clinical manifestations, and socioeconomic status were registered.

**Results:**

In total, 48% of the children (38 from the case group and 10 from the control group) had intestinal parasites. Only four children presented cysts of the *Entamoeba* complex in their samples (three from the case group and one from the control group). By means of PCR, nine samples (9%) amplified for the studied amoebae. In the case group, three (28.13%) amplified for *E. histolytica,* four (30.50%) for *E. dispar,* and one (9.37%) for *E. moshkovskii* while only one (25%) sample amplified for *E. dispar* in the control group.

**Conclusion:**

In general, *E. dispar* predominated. Nevertheless, all those infected with *E. histolytica* were detected within the group of children with diarrhea and we reported the first case of *E. moshkovskii* in the region.

*Entamoeba histolytica* es un parásito protozoario causante de la amebiasis en humanos. Esta infección afecta principalmente a personas que viven en deficientes condiciones de higiene en los países en desarrollo, donde es endémica; los niños menores de cinco años son los más propensos a desarrollarla. La Organización Mundial de la Salud (OMS) estima que, aproximadamente, 500 millones de personas están infectadas con el parásito y el 10 % de ellas presenta amebiasis invasiva. Esta parasitosis es considerada un importante problema de salud pública, pues llega a producir cuadros de disentería amebiana que, de no ser tratados, pueden llevar al paciente a la deshidratación y hasta la muerte ^1^^-^^3^.

Ngobeni, *et al.,* señalan que el término *ameba* engloba las especies pertenecientes a los géneros *Entamoeba, Endolimax* e *lodamoeba;* entre ellas, se destaca *Entamoeba histolytica,* única ameba intestinal que se reconoce como patógena, pues es la causante de la amebiasis. Las restantes especies que pueden encontrarse en la luz del intestino incluyen *Entamoeba dispar, E. moshkovskii, E. bangladeshi, E. hartmannii, E. coli, E. polecki, Endolimax nana* e *lodamoeba butschlii,* consideradas no patógenas ^4^.

Existen otras especies de *Entamoeba,* como *E. gingivalis,* que se encuentra principalmente en la cavidad oral humana, y que también se ha encontrado en Egipto en el aparato genitourinario de usuarias de dispositivos anticonceptivos intrauterinos ^5^, y *E. nuttalli,* que prevalece en primates no humanos, aunque fue detectada en un cuidador de un zoológico en Bélgica ^6^.

Las amebas del género *Entamoeba* comparten muchas características morfológicas y biológicas, pero se caracterizan, en particular, por poseer cromatina adosada a la membrana nuclear interna y presentar dos formas evolutivas durante su ciclo de vida: los trofozoítos o formas vegetativas y los quistes o formas de resistencia ^7^. Los trofozoítos y quistes de *E. histolytica, E. dispar, E. moshkovskii* y *E. bangladeshi* son morfológicamente indistinguibles entre sí y, por consenso, se las ha agrupado en el llamado complejo *Entamoeba*^2^^,^^8^.

El diagnóstico de la amebiasis comúnmente se hace mediante el examen coprológico y la detección al microscopio de quistes o trofozoítos que morfológicamente corresponden a *E. histolytica* o, menos frecuentemente, mediante biopsia de tejido mucoso. Sin embargo, el reconocimiento de *E. dispar* y la detección de *E. moshkovskii* en seres humanos, además del registro de una nueva especie, *E. bangladeshi,* ha complicado el diagnóstico, pues todas son morfológicamente idénticas, aunque diferentes desde el punto de vista genético y bioquímico ^9^^,^^10^.

El examen microscópico de materia fecal presenta serias limitaciones, especialmente, la incapacidad de distinguir entre *E. histolytica, E. dispar, E. moshkovskii* y *E. bangladesí,* y el hecho de que solo puede confirmarse la presencia de *E. histolytica* cuando las muestras presentan trofozoítos hematófagos, situación que es bastante infrecuente ^9^. En este sentido, los métodos moleculares como la PCR han demostrado una mayor sensibilidad de detección comparados con otras técnicas de diagnóstico de la amebiasis, por lo que se ha convertido en la prueba de referencia para el diagnóstico de la amebiasis intestinal ^3^.

No se encontraron publicaciones previas sobre la prevalencia de *E. moshkovskii* en Venezuela y existe muy poca información en cuanto a la prevalencia de *E. histolytica* y *E. dispar* en el país. En ese contexto, el objetivo del presente estudio fue determinar mediante métodos moleculares la frecuencia de estas amebas en niños del municipio Maracaibo, estado Zulia (Venezuela), y determinar la asociación de dicha frecuencia con la presencia o ausencia de diarrea.

## Materiales y métodos

### Diseño del estudio, población y muestra

Se diseñó un estudio de tipo descriptivo, prospectivo y transversal. La población incluyó a todos los niños menores de cinco años con diarrea (área de urgencias) y sin diarrea (triaje de pediatría) atendidos en el Servicio Autónomo Hospital Universitario de Maracaibo, entre enero y julio de 2014.

Se recolectaron 100 muestras de heces frescas de la siguiente manera: 75 de niños menores de cinco años con diarrea aguda, considerados como el grupo de casos, y 25 muestras de heces de niños menores de cinco años sin diarrea, considerados como el grupo de control. Los criterios de inclusión en el grupo de casos fueron: niños menores de cinco años, de cualquier sexo, con diarrea, atendidos en el área de urgencias del Hospital y que no hubieran recibido tratamiento antiparasitario en los dos meses anteriores. Los niños evaluados fueron estratificados por grupos etarios según la clasificación de Quintero ^11^.

Se hizo una encuesta respondida por representantes de todos los niños incluidos en el estudio, para recabar los datos demográficos de edad, sexo y procedencia, y los signos y síntomas de diarrea, presencia de deshidratación, vómito y fiebre. Posteriormente, se hizo la estratificación socioeconómica según Graffar y modificada por Méndez, *et al.*^12^.

### Análisis parasitológico

Se recolectó una muestra fecal por niño. Para ello, a cada representante se le entregó un envase recolector plástico nuevo, limpio, de boca ancha y tapa de rosca (sin preservativos), y se le dieron en forma oral y escrita las recomendaciones necesarias para la correcta recolección de la muestra fecal.

Las muestras fueron trasladadas al Laboratorio de Parasitología de la Escuela de Bioanálisis de la Universidad del Zulia, donde se hizo el examen macroscópico y el microscópico de la muestra fecal, así como la prueba con el método de concentración de formól-éter ^13^. Los resultados del examen coproparasitológico fueron entregados a los representantes de los niños. Una porción de la muestra fue congelada a -20 °C para efectuar posteriormente el análisis molecular (PCR) en el Laboratorio de Biología Molecular del Centro de Investigaciones Endocrino-Metabólicas "Dr. Félix Gómez" con el fin de detectar *E. histolytica, E. dispar* y *E. moshkovskii.*

### Análisis molecular

La extracción y purificación del ADN genómico de *Entamoeba* spp. se llevó a cabo en el Laboratorio de Parasitología de la Escuela de Bioanálisis de la Universidad del Zulia, utilizando un procedimiento estandarizado que incorporó algunos pasos de protocolos de lisis enzimática y choque térmico, y mecánicos, descritos previamente por Rivero, *et al.*^14^.

Todas las reacciones de PCR se llevaron a cabo en un volumen total de 40,0 μl con 8 μl de solución tampón para PCR 10X, 2,4 μl de cloruro de magnesio (MgCl_2_) 25,0 mmol/L; 1 μl de la mezcla de desoxirribonucleótidos (5,0 mmol/L de cada uno); 0,5 μl de GoTaq™ polimerasa de Promega (5 UI/ Ul); 4 μl de la mezcla de oligonucleótidos (50 pmol de cada iniciador) y 15,0 μl del ADN de muestra.

Como controles de referencia, se emplearon la cepa HM-1:IMSS de *E. histolytica,* la cepa SAW760 de *E. dispar,* y la cepa Laredo de *E. moshkovskii,* todas gentilmente donadas por Graham Clark del *London School of Hygiene and Tropical Medicine.* En estos casos, se utilizaron 6 ul de ADN para los pruebas de amplificación por PCR de los controles y, además, se utilizó un control negativo de reacción que solo contenía 1 μl de agua.

Como blanco genómico, se utilizó la secuencia de genes similares al ARNr 16S, con un fragmento específico para el género *Entamoeba* y los fragmentos específicos (iniciadores) para las especies *E. histolytica, E. dispar* y *E. moshkovskii* (Eurofins Genomics) descritos por Khairnar, *et al.*^15^ (cuadro 1). La identidad de las secuencias de oligonucleótidos utilizados y el peso molecular de los fragmentos esperados fueron confirmados en la BLAST (http://www.ncbi.nlm.nih.gov/blast/).

**Cuadro 1:** Oligonucleotides iniciadores utilizados para las especies *Entamoeba histolytica, Entamoeba dispar* y *Entamoeba moshkovskii*

Parásito	Nombre del iniciador	Secuencia del iniciador (5' a 3’)	Tamaño del producto (pb)
*Entamoeba* spp.	E-1	TAAGATGCAGAGCGAAA	≃ 800
	E-2	GTACAAAGGGCAGGGACGTA	
*E. moshkovskii*	Mos-1	GAAACCAAGAGTTTCACAAC	553
	Mos-2	CAATATAAGGCTTGGATGAT	
*E. histolytica*	EH-1	AAGCATTGTTTCTAGATCTGAG	439
	EH-2	AAGAGGTCTAACCGAAATTAG	
*E. dispar*	ED-1	TCTAATTTCGATTAGAACTCT	174
	ED-2	TCCCTACCTATTAGACATAGC	

Se siguió un protocolo de PCR múltiple anidada, estandarizado por los autores del presente estudio para ser efectuado en una sola reacción a partir del protocolo original de Ngui, *et al.*^16^, en el que se hizo el anidado de la región específica para el género *Entamoeba* de genes similares al ARNr 16S. Este incluyó una desnaturalización inicial a 94 °C durante 10 minutos, seguida de 15 ciclos con los siguientes pasos: desnaturalización a 94 °C durante un minuto, apareamiento a 56 °C durante un minuto y extensión a 72 °C durante 1:15 minutos (no se obtuvo el producto para ser verificado en gel de agarosa). En la misma amplificación, se programaron 25 ciclos del siguiente esquema: a 94 °C durante un minuto, a 48 °C durante un minuto, a 72 °C durante 1:15 minutos y, por último, a 72 °C durante 10 minutos. En este paso, se empleó la PCR múltiple para la caracterización genética de *E. histolytica, E. dispar* y *E. moshkovskii,* y se obtuvieron fragmentos de 439 pb, 174 bp y 553 bp. Los tubos Eppendorf se mantuvieron a 4 °C hasta salir del equipo. Todas las muestras fueron corridas en un termociclador Eppendorf (PTC-100 Peltier Thermal Cycler™).

Los productos amplificados se separaron por electroforesis en gel de agarosa al 2,0 % en cámaras horizontales (Bio-Rad Laboratories). Como solución tampón de corrida, se utilizó solución amortiguadora de 89 mM de tris-borato y 2 mM de EDTA, pH: 8 (TBE), la corrida se llevó a cabo a 100 v/ cm durante 30 minutos. Los geles se tiñeron con bromuro de etidio (10 mg/ mi), luego, se visualizaron en un transiluminador ultravioleta (Uvitec) y se fotografiaron con el sistema de fotodocumentación DigiDoc UVP™. Se incluyó un marcador de peso molecular de 100 pb DNA Ladder de Promega.

### Análisis estadístico

La frecuencia de las amebas en relación con las variables estudiadas se expresó con la distribución de frecuencias absolutas y con porcentajes. Para el análisis de los resultados obtenidos, se utilizó el programa estadístico SPSS™, versión 17 (SPSS Inc., Chicago, III, USA). Se empleó la prueba estadística de ji al cuadrado, el test exacto de Fisher o la correlación de Spearman, según correspondiese, para las pruebas de significación estadística entre las variables en estudio. El valor de p<0,05 se aceptó como estadísticamente significativo ^17^.

### Consideraciones éticas

El estudio fue revisado y aprobado por el Comité de Ética del Hospital. Por tratarse de una investigación no terapéutica en la que se emplearon muestras fecales de niños menores, los padres autorizaron la entrega de la muestra mediante un consentimiento informado y dieron la información necesaria para la encuesta. Se guardó en absoluta confidencialidad la información de los pacientes.

## Resultados

La mediana de edad de los niños estudiados fue de 3 ± 1,093 años, siendo el menor de cinco meses y el mayor de cinco años. En total, participaron 38 niñas (31 en el grupo de casos y siete en el grupo de control) y 62 varones (42 en el grupo de casos y 18 en el grupo de control). El 48 % de los niños (38 del grupo de casos y 10 del grupo de control) presentaron enteroparásitos según el examen microscópico directo o por concentración. Mediante este procedimiento, se detectaron quistes del complejo *Entamoeba* en las muestras fecales de tres (4,6 %) individuos del grupo de casos y de uno (7,1 %) del grupo de control.

**Figura 1:**
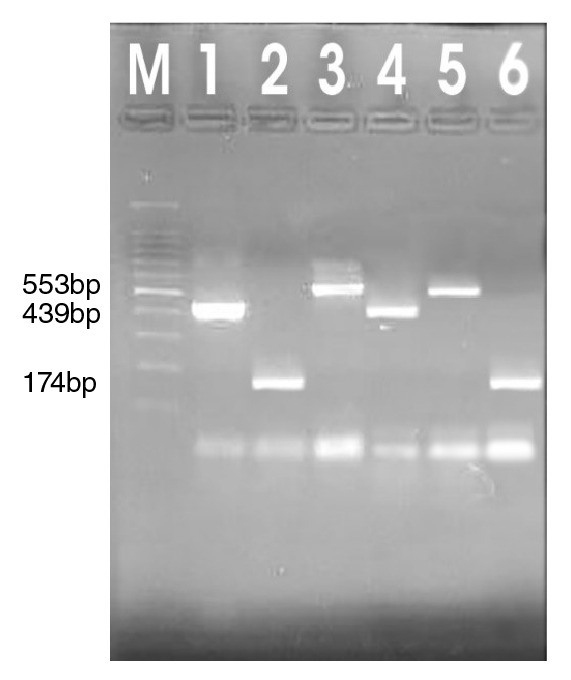
Identificación por PCR de *Entamoeba histolytica, Entamoeba dispar* y *Entamoeba moshkovskii*

Como resultado de la PCR aplicada a las 100 muestras, se amplificaron nueve muestras biológicas (figura 1), distribuidas de la siguiente manera: tres muestras con £ *histolytica,* cinco con *E. dispar* y una con *E*. *moshkovskii.*

M: marcador de peso molecular. 1: control de referencia de *Entamoeba histolytica* (HM-1: IMSS). 2: control de referencia de *Entamoeba dispar* (SAW 760). 3: control de referencia de *Entamoeba moshkovskii* (cepa Laredo). 4, 5 y 6: ADN extraído de muestras de heces de pacientes positivos para cada una de las amebas estudiadas

En el cuadro 2 se presentan los resultados de la PCR según los grupos de casos y controles. Se encontraron ocho muestras positivas en el grupo de casos, tres correspondían a *E. histolytica,* cuatro a *E. dispar* y una a *E. moshkovskii,* en tanto que, en el grupo de control, solo se detectó un individuo con *E. dispar;* no hubo casos de infección simultánea por dos o más especies de las amebas estudiadas entre los niños evaluados.

**Cuadro 2:** Resultados de la PCR por especie de *Entamoeba* en el grupo de casos y en el de control

Especies parasitarias	Grupo estudiado	
	Casos	Control
	n	n
*Entamoeba histolytica*	3	0
*Entamoeba dispar*	4	1
*Entamoeba moshkovskii*	1	0
Total	8	1

Los parásitos que se encontraron simultáneamente con *E. histolytica* fueron los helmintos *Ascaris lumbricoides* y *Trichuris trichiura,* pero no se la encontró asociada con ningún protozoario intestinal. En los niños con *E. dispar,* se presentó la mayor cantidad de asociaciones. Se observó en conjunto con *Blastocystis* sp. (16,6 %), con protozoarios como *E. coli* (33,3 %), *E. nana* (16,6 %) y *Giardia lamblia* (16,6 %), y con helmintos como *A. lumbricoides* (16,6 %). En el caso de *E. moshkovskii,* esta no se observó asociada a ningún otro parásito. Se detectaron infecciones exclusivas por *E. histolytica* y *E. dispar,* un caso de cada una, sin asociación con ningún helminto o protozoario intestinal.

Mediante el examen microscópico solo se observaron cuatro muestras positivas para el complejo *Entamoeba,* todas pertenecientes al grupo de preescolares. Una vez efectuado el procesamiento por PCR, se detectó un mayor número de casos, con nueve niños positivos y presencia de estas amebas tanto en el grupo de lactantes mayores como en el de lactantes menores, además de algunos casos más entre los mismos preescolares. En general, se encontró un predominio de las amebas en el grupo de niños entre dos y cinco años, con 3 % para *E. histolytica* y 4 % para *E. dispar.* Apenas un caso de *E. dispar* se detectó en lactantes mayores. Cabe destacar que el único caso de *E. moshkovskii* correspondía a un lactante menor (seis meses). No se encontró una diferencia significativa en esta variable (p>0,05).

En cuanto a la prevalencia de las amebas por sexo, se apreció que la mayoría de los casos de infección por *E histolytica, E. dispar* y *E. moshkovskii* se detectó en el sexo masculino, aunque no se determinó una diferencia significativa (p>0,05).

En cuanto a las variables de prevalencia de las amebas y el estrato socioeconómico de los niños, se evidenció que todos los casos de infección por *E. histolytica* correspondían a niños en situación de pobreza crítica, en tanto que los casos de *E. dispar* se encontraron repartidos entre los dos estratos más pobres de la escala (pobreza relativa y crítica), y el único caso de *E. moshkovskii* detectado pertenecía el estrato de pobreza relativa.

## Discusión

La mayoría de los estudios epidemiológicos sobre la infección por *E. histolytica* se desarrollaron antes de la descripción de las especies del complejo *Entamoeba.* Por ello, existe una clara necesidad de desarrollar estudios epidemiológicos para distinguir entre las especies de amebas y determinar la verdadera prevalencia de la infección por *E. histolytica.* En el presente estudio, se lograron identificar mediante PCR *E. histolytica, E. dispar* y *E. moshkovskii* en las heces de niños menores de cinco años con y sin diarrea, atendidos en el Servicio Autónomo Hospital Universitario de Maracaibo.

En las muestras analizadas, predominó la especie *E. dispar* (cinco muestras), seguida de *E. histolytica* (tres muestras). Estos resultados coinciden con el estudio previo de Bracho, *et al.*^18^, en niños con diarrea atendidos en el Hospital, en el que predominaron las infecciones por *E. dispar.* Estos autores refieren que, en seis muestras, identificaron ADN de *E. dispar* y, en dos, ADN de *E. histolytica,* y en ninguna hubo asociación entre las amebas estudiadas.

En un estudio en comunidades rurales al noreste de Suráfrica, se registraron prevalencias de 4,1 % de *E. histolytica,* de 14,7 % de *E. dispar* y de 15,9 % de *E. moshkovskii*^19^. Sharbathkori, *et al.*^20^, determinaron la prevalencia de *Entamoeba* spp. en niños con disentería de Irán y encontraron *E. histolytica* y *E. dispar* en dos (2/25) y tres (3/25) muestras, respectivamente. Asimismo, en Colombia, López, *et al.*^21^, hicieron estudios para diferenciar el complejo *E. histolytica-E. dispar* mediante la detección de Gal-GalNAc-lectina y PCR. Sus resultados arrojaron una prevalencia de 0,6 a 1,4 % para *E. histolytica* y de 15 a 17 % para *E. dispar.* En múltiples estudios basados en pruebas de biología molecular a nivel internacional, se señala el predominio de las infecciones por *E. dispar* frente a las de *E. histolytica*^8^^,^^22^^-^^25^.

Algunos estudios en la población general a nivel nacional e internacional contrastan con estos resultados. Es el caso de Mora, *et al.*^26^, quienes establecieron la prevalencia de *E. histolytica* y *E. dispar* en pacientes con diarrea de Cumaná (Venezuela), mediante PCR, y reportaron una prevalencia de *E. histolytica* de 6,3 % y de *E. dispar* de 4,44 %, detectando cuatro casos de infecciones mixtas. Los más afectados por ambas especies fueron niños y jóvenes. Ngui, *et al.*^16^, estudiaron comunidades rurales de Malasia y refieren que la infección con *E. histolytica* (75,0 %; 39/52) fue la más común, seguida de *E. dispar* (30,8 %; 18/52) y *E. moshkovskii* (5,8 %; 3/52). De estos casos, 33 (63,5 %) individuos presentaban solo *E. histolytica,* 10 (19,2 %) portaban exclusivamente *E. dispar* y tres (5,8 %) solo *E. moshkovskii;* seis muestras (11,5 %) presentaron infección mixta de *E. histolytica* y *E. dispar.* Asimismo, Roshdy, *et al.*^27^, mediante PCR múltiple anidada y PCR en tiempo real de pacientes con disentería en un hospital del Cairo, Egipto, encontraron 10,3 % de prevalencia de *E. histolytica* y 8,7 % de *E. dispar,* y no detectaron casos de *E. moshkovskii.*

En el presente estudio, solo se detectó un caso de *E. moshkovskii* en un niño de seis meses de edad con diarrea. Es importante resaltar que este es el primer informe de *E. moshkovskii* en niños de la región zuliana y en el país. El primer reporte de esta ameba en humanos fue recogido por Haque, *et al.*^28^, en una niña de Bangladesh. Estudios posteriores en ese mismo país revelaron una prevalencia de *E. moshkovskii* del 21 % en niños de dos a cinco años mediante herramientas moleculares ^29^.

En Latinoamérica hay muy pocas referencias sobre la detección de *E. moshkovskii* en humanos; solo en Ecuador, Colombia, Brasil y Venezuela se han hecho investigaciones sobre esta especie. Levecke ^30^ refiere que, de 674 muestras de una comunidad rural al sur de Ecuador, solo 101 contenían quistes del complejo *Entamoeba;* sin embargo, mediante PCR-RLHB se detectaron 22,8 % casos de *E. dispar,* en tanto que no se encontraron infecciones por *E. histolytica* ni *E. moshkovskii.* Bachkanji ^31^ evaluó mediante PCR 150 muestras de niños de 0 a 10 años del estado Sucre (Venezuela), que al microscopio resultaron positivas para el complejo *Entamoeba,* en tanto que utilizando herramientas moleculares el 19,30 % de ellas amplificó para *E. histolytica,* el 4 % para *E. dispar* y el 4,70 % presentó asociación de ambas amebas, pero no se detectó *E. moshkovskii.*

En el estudio de López, *et al.*^32^, en un área rural del centro de Colombia, se encontró mediante PCR múltiple una frecuencia del 49,1 % (89/181); al diferenciar por especie, el 23,2 % (42/181) de las muestras fue positivo para *E. dispar,* el 25,4 % (46/181), para *E. moshkovskii* y, el 0,55 % (1/181), para *E. histolytica;* además, se observaron infecciones mixtas de *E. dispar* y *E. moshkovskii* en 4,42 % (8/181) de las muestras. Asimismo, Soares, *et al.*^33^, estudiaron pacientes que asistían a un sistema de salud pública en Bahía (Brasil), mediante técnicas moleculares e inmunológicas. Por microscopía, se encontró una prevalencia de 0,49 % del complejo *Entamoeba* en 273 de 55.218 pacientes. De estos 273 individuos con microscopía positiva, solo 90 aceptaron participar en el estudio, entre los cuales, el 8,9 % (8/90) fueron positivos para *E. histolytica* por serología. En las muestras analizadas por PCR, el 80 % (72/90) fueron positivas para *E. dispar,* aunque no fue posible identificar *E. histolytica* ni *E. moshkovskii* y, en el resto de las muestras (18/90), no hubo amplificación. Puede concluirse entonces que, de los países mencionados, solo en Colombia se había reportado la presencia de *E. moshkovskii.*

Es importante mencionar el estudio realizado por Ngui, *et al.*^34^, en el cual se examinaron 504 muestras fecales de humanos y perros mediante microscopía y PCR en Malasia, se encontró que la especie más común era *E. dispar* (26,5 %; 13/49), seguida por *E. histolytica* y *E. moshkovskii* (con 20,4 % para cada especie). En los animales, *E. moshkovskii* (46,7 %) fue la especie más común, seguida por *E. histolytica* y *E. dispar,* con 20,0 % y 13,3 %, respectivamente. Todo ello demuestra la presencia de la especie patógena de *Entamoeba* en perros, los que podrían ser un reservorio o un huésped mecánico para la amebiasis humana.

Al evaluar la presencia de las amebas en los dos grupos estudiados, se encontró que *E. histolytica* solo se detectó en el grupo de niños con diarrea y no en el grupo control. *E. dispar,* considerada como una especie no patógena, sí se encontró en ambos grupos de estudio, aunque en porcentajes diferentes. El único caso de *E. moshkovskii* también fue detectado en el grupo de niños con diarrea.

A pesar de que el número de individuos estudiados en el grupo control fue menor, es importante señalar que *E. histolytica* solo se detectó en el grupo de casos. Si bien la literatura científica refiere la posibilidad de individuos asintomáticos con *E. histolytica* (portadores asintomáticos) ^35^^-^^37^, en el presente estudio no se detectó esta situación, pues todos los pacientes infectados presentaban diarrea. Es innegable la capacidad patogénica de *E. histolytica,* como se ha demostrado en múltiples publicaciones ^38^^-^^41^, y en este caso, la coincidencia del hallazgo del parásito y la diarrea confirman esta premisa. Samie, *et al.*^19^, refieren que solo *E. histolytica* estuvo asociada estadísticamente con diarrea en su estudio sobre la distribución de *Entamoeba* en comunidades rurales de Suráfrica. La diarrea en los niños infectados por *E. dispar* y *E. moshkovskii* podría explicarse por la asociación con otros enteroparásitos que la producen (por ejemplo, *G. lamblia),* situación observada en el presente estudio.

Los estudios de Ugboko, *et al.*^42^, indican que las enfermedades diarreicas parasitarias de importancia para la salud pública son la amebiasis *(Entamoeba histolytica),* la criptosporidiosis *(Cryptosporidium* spp.) y la giardiasis *(G. lamblia),* lo que puede comprobarse por el predominio de las amebas encontradas en este estudio en el grupo de niños con diarrea, a pesar de que no todas las amebas identificadas se consideran patógenas.

Mediante el examen microscópico, solo se observaron cuatro muestras positivas para el complejo *Entamoeba,* todas en el grupo de preescolares. Una vez efectuado el procesamiento por PCR, se logró evidenciar un mayor número de casos, resultando positivos nueve niños, con casos tanto en el grupo de lactantes mayores como en el de lactantes menores, además de algunos más dentro del grupo de los preescolares, lo que era predecible, pues la detección del ADN parasitario mediante PCR es mucho más sensible que la microscopía ^3^^,^^8^^,^^27^^,^^33^.

En cuanto a la presencia de las amebas según la edad, en general, se encontró un predominio en el grupo de los preescolares, con 3 % para *E. histolytica* y 4 % para *E. dispar.* Sin embargo, no se evidenció una diferencia estadísticamente significativa entre las variables.

Los resultados del presente estudio difieren de los de Rivero, *et al.*^14^, en individuos de una comunidad del estado Zulia, en el que se detectaron casos de estas amebas en los menores de dos años, lo que pudiera explicarse por los cuidados maternos que reciben estos niños, así como por el efecto protector contra *E. histolytica* que confieren el calostro y la leche materna en los lactantes ^43^^,^^44^.

Se observó una total congruencia entre los resultados microscópicos del complejo *Entamoeba* y los de la PCR. Los cuatro casos detectados mediante microscopía también fueron positivos por PCR, y fueron dos de *E. histolytica* y dos de *E. dispar.* Por supuesto, dado que la PCR es un método más sensible y específico, con ella se detectaron cinco casos más que no habían sido diagnosticados mediante el examen microscópico. Esto difiere de lo informado por Bracho, *et al.*^18^, quienes no observaron coincidencia entre los resultados del examen directo de heces y los resultados por PCR en niños del Servicio Autónomo Hospital Universitario de Maracaibo. En este estudio, mediante el examen microscópico no se detectaron muestras con *E. histolytica* y *E. dispar,* en tanto que mediante PCR, ocho muestras amplificaron para alguna de las especies de amebas. Los autores consideraron factible que las formas evolutivas de dichas amebas se encontraran en muy poca cantidad o parcialmente destruidas, por lo que no pudieron ser detectadas mediante microscopía y sí mediante PCR, que es una técnica mucho más sensible.

Aunque las especies estudiadas se encontraron en los niños pertenecientes a los estratos de pobreza relativa y pobreza crítica, el mayor porcentaje (seis casos) se encontró entre aquellos en situación de pobreza crítica. Esto permite relacionar la presencia de estas amebas, especialmente la patógena *E. histolytica,* con las peores condiciones de vivienda de todos los estratos, los ingresos más bajos y el menor grado de instrucción de los padres de familia. Se hace hincapié en las condiciones de la vivienda, principalmente, ya que son las más relacionadas con las condiciones sanitarias. Según la escala de Graffar, en el estrato V, el tipo de vivienda corresponde a un rancho con condiciones sanitarias marcadamente inadecuadas. Estos resultados coinciden con los de otros estudios a nivel nacional e internacional ^25^^,^^34^^,^^45^^-^^47^^)^ en los que se ha encontrado que los parásitos intestinales están asociados principalmente a las malas condiciones higiénicas, el escaso saneamiento del entorno y la precariedad socioeconómica de las familias.

En conclusión, predominó la especie *E. dispar* entre los niños estudiados; sin embargo, los casos de *E. histolytica* se detectaron solo en el grupo de niños con diarrea y en situación de pobreza crítica. Además, se encontró la primera evidencia de infección por *E. moshkovskii* en humanos en Venezuela, lo que requiere la continuación de estudios similares en otras comunidades.
